# Knowledge-guided contextual gene set analysis with large language models

**DOI:** 10.1093/bioinformatics/btag214

**Published:** 2026-07-07

**Authors:** Zhizheng Wang, Chi-Ping Day, Chih-Hsuan Wei, Qiao Jin, Robert Leaman, Yifan Yang, Shubo Tian, Aodong Qiu, Yin Fang, Qingqing Zhu, Xinghua Lu, Zhiyong Lu

**Affiliations:** Division of Intramural Research (DIR), National Library of Medicine (NLM), National Institutes of Health (NIH), Bethesda, MD 20892, United States; Cancer Data Science Laboratory, Center for Cancer Research, National Cancer Institute (NCI), National Institutes of Health (NIH), Bethesda, MD 20892, United States; Division of Intramural Research (DIR), National Library of Medicine (NLM), National Institutes of Health (NIH), Bethesda, MD 20892, United States; Division of Intramural Research (DIR), National Library of Medicine (NLM), National Institutes of Health (NIH), Bethesda, MD 20892, United States; Division of Intramural Research (DIR), National Library of Medicine (NLM), National Institutes of Health (NIH), Bethesda, MD 20892, United States; Division of Intramural Research (DIR), National Library of Medicine (NLM), National Institutes of Health (NIH), Bethesda, MD 20892, United States; Division of Intramural Research (DIR), National Library of Medicine (NLM), National Institutes of Health (NIH), Bethesda, MD 20892, United States; Department of Biomedical Informatics, School of Medicine, University of Pittsburgh, Pittsburgh, PA 15206, United States; Division of Intramural Research (DIR), National Library of Medicine (NLM), National Institutes of Health (NIH), Bethesda, MD 20892, United States; Division of Intramural Research (DIR), National Library of Medicine (NLM), National Institutes of Health (NIH), Bethesda, MD 20892, United States; Department of Biomedical Informatics, School of Medicine, University of Pittsburgh, Pittsburgh, PA 15206, United States; Division of Intramural Research (DIR), National Library of Medicine (NLM), National Institutes of Health (NIH), Bethesda, MD 20892, United States

## Abstract

**Motivation:**

Gene set analysis (GSA) is a foundational approach for interpreting genomic data of diseases by linking genes to biological processes. However, conventional GSA methods overlook clinical context of the analyses, often generating long lists of enriched pathways with redundant, nonspecific, or irrelevant results. Interpreting these requires extensive, ad-hoc manual effort, reducing both reliability and reproducibility.

**Results:**

We introduce cGSA, a novel AI-driven framework that enhances GSA by incorporating context-aware pathway prioritization. cGSA integrates gene cluster detection, enrichment analysis, and large language models to identify pathways that are not only statistically significant but also biologically meaningful. Benchmarking on 102 curated gene sets across 19 diseases and ten disease-related biological mechanisms shows that cGSA outperforms baseline methods by over 30%, with expert validation confirming its increased precision and interpretability. Two independent case studies in melanoma and breast cancer further demonstrate its potential to uncover context-specific insights and support targeted hypothesis.

**Availability and Implementation:**

The demo website is publicly available at https://www.ncbi.nlm.nih.gov/CBBresearch/Lu/Demo/cGSA/, while the data and code can be accessed at https://github.com/ncbi-nlp/cGSA.

## 1 Introduction

Genomic, epigenetic, and transcriptomic analyses have greatly advanced our understanding of biological mechanisms involved in normal organism development and disease pathogenesis (Fatumo et al. 2022, Zhou et al. 2024). These analyses generate data for the expression, regulation, or alteration of genes, so the results often include gene sets. For instance, exome sequencing allows mutation calling in coding genes (Hoang et al. 2013, Warr et al. 2015), RNA sequencing reads gene expression levels (Pickrell et al. 2010, Parekh et al. 2018), and ATAC-seq (i.e. assay for transposase-accessible chromatin sequencing) identifies transcriptionally active genes (Kumar et al. 2020).

Functional interpretation of such gene sets is central to genomic research, enabling scientists to formulate hypotheses for disease mechanisms, potential biomarkers, or therapeutic approaches (Zhou et al. 2013, Ma et al. 2024). Gene set analysis (GSA) (Subramanian et al. 2005, Liu et al. 2023) is a widely used approach, which offers critical insights into the molecular mechanisms and biological processes underlying gene sets. Such mechanistic insights are generally crucial for uncovering disease therapeutic targets. Over its development, GSA has progressed through two notable methodological paradigms: classical functional enrichment analysis and emerging solutions based on large language models (LLMs). Traditional GSA methods (Kuleshov et al. 2016, Raudvere et al. 2019) typically compare gene sets against predefined categories within manually curated databases, making them applicable to high-throughput omics datasets by identifying functions that are statistically enriched. Recent LLM-based GSA methods (Hu et al. 2025, Wang et al. 2025) leveraging instruction-based learning and self-verification strategies have demonstrated promise by generating meaningful functional annotations and coherent interpretations.

However, these classical or LLM-based GSA methods do not include experimental contexts or study objectives associated with the research topic, often producing generic, fragmented, or redundant results. This issue is particularly pronounced in high-throughput omics of disease-specific studies, where the context relevance of results is highly sensitive to research topics (Mooney et al. 2014). Lacking mechanisms for prioritizing contextually relevant results, researchers frequently encounter large lists of pathways or functions enriched from gene sets. Consequently, results are often filtered in an ad-hoc manner, such as only selecting statistically top-ranked pathways, which in the risk of overlooking contextually relevant functional terms that are buried at the bottom of the list.

Furthermore, current GSA methods typically overlook gene interactions and regulatory relationships within gene sets (Peng et al. 2021), resulting in central hub genes over-dominating the enrichment results regardless of their mechanistic involved in the required context. For example, the gene TP53 is frequently identified as very significantly enriched across numerous cancer-related pathways, even when many of these pathways are not directly relevant to the required cancer type. Such a bias not only inaccurately inflates the significance of hub genes but also limits the discovery of functionally relevant pathways that may be critical to the research objectives yet remain underexplored.

To overcome these limitations, we presented contextualized Gene Set Analysis (cGSA), a novel LLM-based pipeline that integrates both domain knowledge and research context into the reasoning process for gene set enrichment analysis. Specifically, cGSA features three key technical innovations:

- **Context-aware GSA.** Incorporates contextual information to tailor gene set analysis to specific study objectives and experimental settings, improving relevance and accuracy while reducing background noise for domain-expert review.
- **Gene network clustering.** Clusters genes using protein interaction networks and community detection algorithms, enhancing pathway specificity and mitigating inflated enrichment driven by central hub genes.
- **Interpretable results.** Guides LLMs to generate concise, biologically meaningful, and contextually relevant pathway summaries through evidence-based reasoning, minimizing hallucinations and overgeneralization.

Moreover, to fill the gap that current benchmarks used in existing GSA methods merely include co-expressed genes, we manually created a new benchmark dataset of differentially expressed genes (DEGs) with explicit contextual information, which is central to enrichment analysis. To assess the real-world utility of cGSA, we further applied the workflow to two practical research studies: (1) development of resistant to immune checkpoint blockade in melanoma and (2) cell-cell communication within tumor microenvironment of breast cancer. Expert evaluation confirms that cGSA enhances functional interpretations by aligning with established findings or making connections with contextually relevant knowledge.

## 2 Method

**Figure 1:**
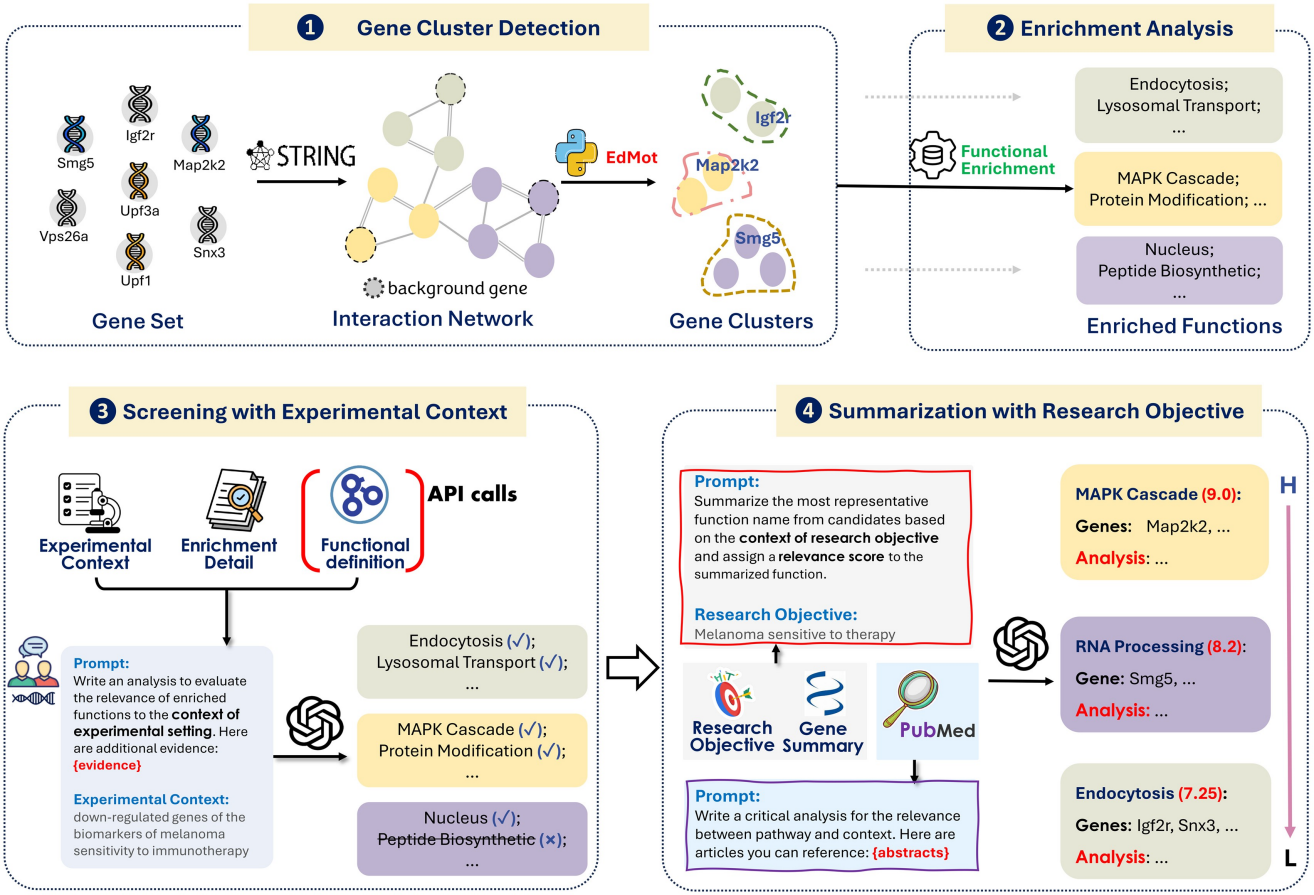
The cGSA workflow, which integrates multiple computational methods to identify contextually relevant pathways from input gene sets. It consists of four key steps: *Gene Cluster Detection* groups the input DEGs into distinct communities using the STRING database and the EdMot algorithm. *Enrichment Analysis* identifies the most significant pathways (*P*-value<0.001) for gene clusters by querying established target databases. *Pathway Screening* employs the large language model to filter enriched pathways based on the experimental context where the DEGs are derived. *Pathway Summarization* selects the most representative pathway for each gene cluster by integrating filtered pathway candidates with the overarching research objective and gene summaries. Final pathway outputs are ranked by relevance scores, from high (H) to low (L).

As shown in Fig. 1, cGSA is an LLM-based GSA pipeline that comprises four steps to identify context-aware functional pathways (*P*) from the DEGs. It explores significantly enriched pathways grounded in domain-specific databases (*DB*), while aligning with specific experimental settings ($C_{E}$) and research objectives ($C_{R}$). Meanwhile, by integrating multiple biological statistical tools (Δ), cGSA enhances the reliability of pathway prioritization and summarization. The process is formally defined as follows.


(1) $$P_{i=1}^{K}=\mathrm{cGSA}(\mathrm{DEG},C_{E},C_{R},DB,\Delta )$$

### 2.1 Gene cluster detection

The first step performs graph-based clustering of the input DEGs to partition them into non-overlapping communities. This procedure identifies gene subsets that reflect coherent regulatory or functional structure, such as co-regulated modules or shared biological processes, reducing the over-domination of central hub genes. By explicitly modeling gene interactions, it captures higher-order organizational patterns that are typically not detected by classical GSA methods.

Specifically, given a set of DEGs, cGSA constructs a gene interaction network G=(V,E) using the STRING database,^1^ where $V=V^{i}\cup V^{o}$ denotes genes derived from DEGs ($V^{i}$) and those present in STRING but not included in the input DEGs ($V^{o}$). The E represents pairwise interactions. cGSA then applies the EdMot algorithm (Li et al. 2019), a motif-aware community detection method, to split G as non-overlapping communities $c_{i}$, enabling the identification of functionally coherent gene modules that reflect underlying biological patterns.


(2) $$C=\overset{K}{\underset{i=1}{\cup }}c_{i}(v_{i},e_{i})=\mathrm{EdMot}(G,\Theta )$$

*K* denotes the number of clusters and Θ represents the set of EdMot hyperparameters, including the number of components [set to 3 according to Li et al. (2019)] and the motif cutoff threshold (set to 150 based on the average degree of PPI network).

### 2.2 Enrichment analysis

Building on the gene clusters C, this stage identifies statistically enriched functions for each cluster to support downstream contextual analysis. Through the classical enrichment analysis, cGSA provides reliable candidate functional terms to the LLM, rather than requiring de novo generation, thereby reducing hallucinations.

Specifically, for each cluster $c_{i}=(v_{i},e_{i})\in C$, cGSA submits the gene set $v_{i}$ and the user-specified curated databases to the Enrichr API^2^ for gene set enrichment analysis. Each cluster is then assigned a set of significantly enriched terms *T* (e.g. pathways) with associated *P*-values, formally defined as


(3) $$c_{i}^{T}=\text{Enrichr}(v_{i};db,p),$$

to ensure the significance of enriched pathways in *T*, we only retain the functions with a *P*-value < 0.001. The database set db∈DB is selected from Enrichr’s backend resources to match the experimental context.

### 2.3 Pathway screening

Since each $c_{i}^{T}$ may be extensive, this stage incorporates user-provided experimental context, such as treatment conditions or biological interests, to prioritize pathways relevant to the context and reduce the candidate space.

Specifically, given contextual information $C_{E}$, cGSA builds an evidence-grounded prompt (Supplementary Appendix A.1) for the LLM to assess the relevance of pathways in $c_{i}^{T}$ with respect to $C_{E}$. As supporting evidence, cGSA first collects the statistical information (I) from classical enrichment analysis to indicate the enrichment significance of pathways, including *P*-values and overlapping genes. It further reveals formal functions (F) of pathways by calling the OLS API^3^ to retrieve the GO database. Based on the user-provided contexts and collected evidence, the screening process can be defined as follows.


(4) $$S_{c_{i}^{T}}=\text{LLM}(c_{i}^{T},C_{E},I,F),$$

### 2.4 Pathway summarization

Pathway summarization aims to identify the most representative pathway from the screened candidates for each gene cluster while further mitigating hallucination risks through context-aware synthesis.

In this stage, the LLM is instructed to generate a prominent pathway ($P_{c_{i}^{T}}$) from $S_{c_{i}^{T}}$ based on the input research objectives $C_{R}$, which provide domain-specific background and analytical goals. To ensure that the summarized pathway is consistent with underlying gene functions, cGSA incorporates gene-level summaries S derived from each cluster as additional supporting evidence for pathway validation. Furthermore, following prior work by Hu et al. (2025), the LLM also assigns a relevance score *R* between 0.0 and 10.0 to each summarized pathway to enhance interpretability (Supplementary Appendix A.2). This process can be formally expressed as follows.


(5) $$P_{c_{i}^{T}},R=\text{LLM}(S_{c_{i}^{T}},C_{R},S).$$

Notably, all prompts remain invisible to the user, meaning that cGSA constitutes an end-to-end automated workflow. Additionally, cGSA queries PubMed to generate transparent explanations that align the summarized pathways $P_{c_{i}^{T}}$ with the research objectives $C_{R}$.

## 3 Results

We conducted a comprehensive evaluation to examine the advantages of cGSA and its ability to overcome the limitations of traditional enrichment analysis and LLM-based methods. This evaluation is essential for verifying whether cGSA reliably generates contextually relevant and biologically meaningful pathways while minimizing irrelevant outputs.

### 3.1 Dataset

We curated 102 DEG sets as shown in Table 1 from 31 disease-related articles that are selected from a pool of 331 studies published between 1st October 2022 and 30nd June 2024. These articles contained research objectives, experimental designs, function annotations, and DEG lists. All genes were normalized to NCBI gene identifiers (Supplementary Appendix B).

**Table 1:** Statistic of DEGs used for evaluating cGSA.

	DEGs	Avg. Genes	Dis^a^	Mech^b^	Species
PubMed	102	913.6	19	10	Human & Mouse
Melanoma	6	757.5	1	–	Mouse
TME^c^	3	107.3	–	1	Human

a Abbreviation of “Disease.”

b Abbreviation of “Mechanism.”

c Abbreviation of “tumor microenvironment.”

**Figure 2:**
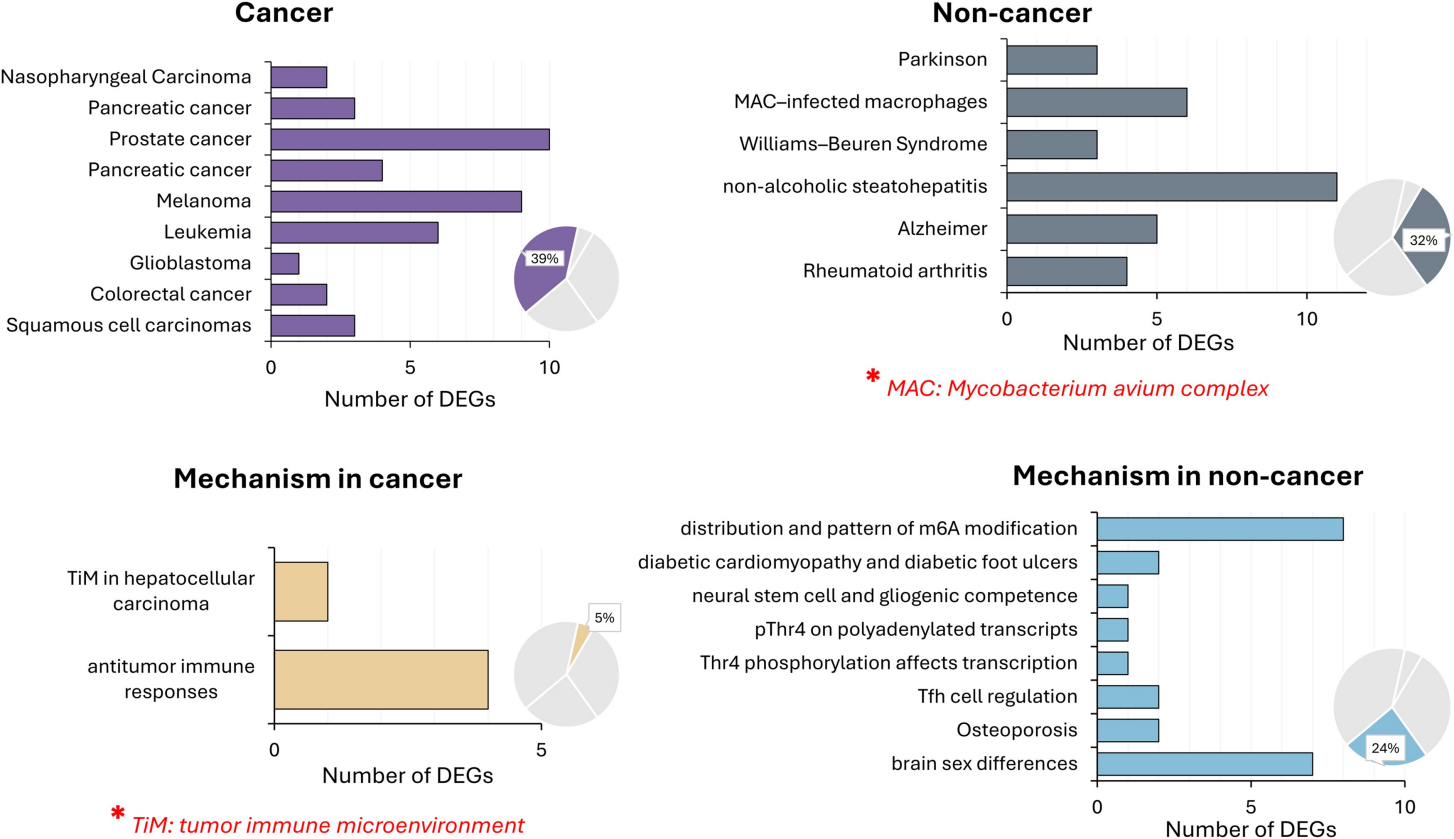
Detailed distribution of DEGs across different disease categories.

The curated DEG sets span 19 cancer-related diseases (e.g. melanoma) and ten disease-associated biological mechanisms (e.g. antitumor immune response). As shown in Fig. 2, 39% of the DEG sets are associated with cancer-related diseases and 5% with cancer-related biological mechanisms, whereas 32% are associated with non-cancer diseases and 24% with non-cancer biological mechanisms. Each DEG set is derived directly from the original studies and associated with well-defined contextual biological functions. The functional annotations are treated as ground truths, while the contextual annotations provide essential information for interpreting differential gene expression under specific biological or experimental conditions.

Additionally, we include two real-world DEGs to demonstrate the practical applicability of cGSA: one derived from preclinical experiments investigating the development of resistance in melanoma, and another collected from open data on cell–cell communication within the tumor microenvironment of breast cancer.

### 3.2 Experimental setting and baselines

In cGSA, we used GPT-4o (version 20240513) as the backend LLM for the pathway screening and summarization steps. The gene interaction network was constructed using the STRING API (version 12.0), and traditional enrichment analysis was performed using the Enrichr API (version 20230608).

We compared cGSA with five baseline methods, including three advanced LLMs: GPT-4o (version 20240513), GPT-4 (version 20230613), and Llama 3.1-70B (version 20240418), and two gene set enrichment analysis tools, g: Profiler and Enrichr. For fair comparisons, Enrichr configured the same databases as cGSA, while g: Profiler relied on databases specified in its original publication. The LLM-based GSA baselines followed the instruction proposed by Joachimiak et al. (2024) to summarize plausible biological terms for each gene set. Importantly, all comparison methods only take the DEGs as input and none of them incorporates contextual information.

The evaluation for cGSA was conducted using the harmonic mean of accuracy (ACC) and hit ratio (HIT). HIT measures the proportion of ground-truth pathways covered by the cGSA outputs, while ACC quantifies the proportion of output pathways that align with the ground truths. In addition to the automatic evaluation, two independent domain experts performed blind annotations of the results.

### 3.3 cGSA outperforms baselines by harnessing contextual information

cGSA generates pathway names in free text, which could be semantically aligned with ground-truth annotations without requiring exact lexical matches. For example, the output of cGSA is “glycine cleavage system,” whereas the corresponding GO annotation is “glycine decarboxylation via glycine cleavage system.” Although not identical, they exhibit high semantic similarity. Therefore, to enable a more meaningful evaluation, we employ semantic similarity scores to assess the alignment between predicted pathways and ground-truth annotations. Specifically, such semantic similarity is computed as the cosine similarity between embedding vectors of predicted terms and ground truths, obtained using MedCPT that is a state-of-the-art biomedical semantic encoder.

**Figure 3:**
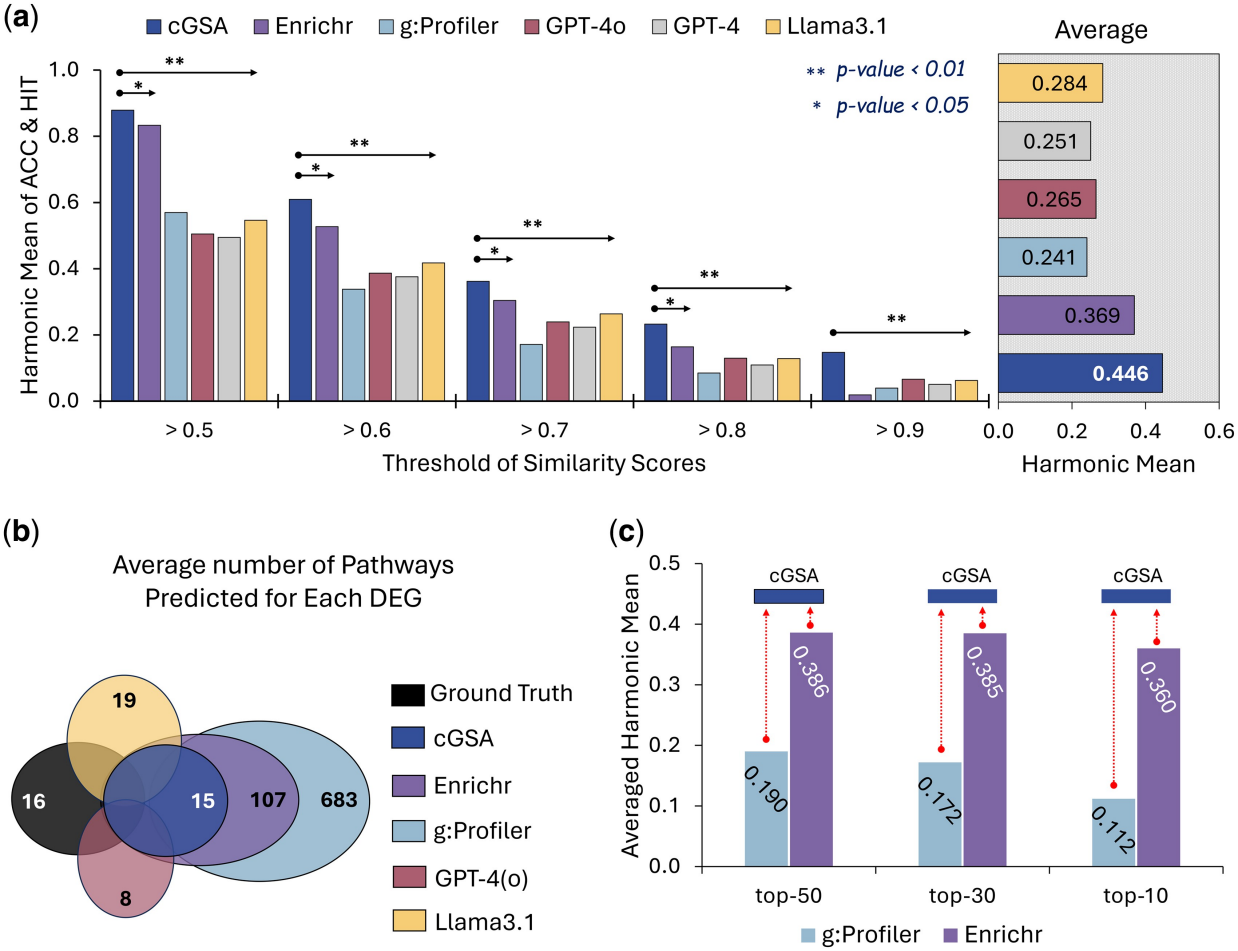
cGSA outperforms existing approaches by a significant margin. (a) The harmonic mean value achieved by cGSA and other baseline methods across different similarity score thresholds. The *P*-values are calculated by a one-tailed paired *t*-test with 95% confidence intervals for 102 DEGs. The average values across all thresholds are presented in the right panel. (b) The average number of pathways identified by different methods for each input DEGs. GPT-4(o) indicates that both GPT-4 and GPT-4o produced the same average number of pathways per DEGs. (c) Performance comparison between cGSA and the top K (K = 10, 30, 50) results from conventional functional enrichment analysis tools (i.e. g: Profiler and Enrichr). The similarity score threshold is set to 0.5.

The results of cGSA and baseline methods are summarized in Fig. 3. At a similarity score threshold of 0.5, cGSA achieves a harmonic mean value of 0.879, representing a 5% improvement over Enrichr, 30% over g: Profiler, 33% over Llama 3.1, and 37% over GPT-4o. Across thresholds ranging from 0.5 to 0.9, cGSA maintains an average harmonic mean value of 44.6%, which significantly outperforms the baseline methods (Fig. 3a, right). These outcomes highlight cGSA’s ability to generate pathways that are more closely aligned with ground truths by generating fewer but more precise outputs.

As shown in Fig. 3b, cGSA generated 15 pathways per DEG on average, in contrast to 107 pathways predicted by Enrichr and 683 pathways predicted by g: Profiler. Despite a substantial reduction in the number of predicted pathways, cGSA achieved a HIT rate 21% higher than g: Profiler and is comparable to Enrichr (Supplementary Appendix B), indicating that it successfully recalled more ground-truth pathways while maintaining a conservative output. So, cGSA demonstrated a significantly higher accuracy than both g: Profiler and Enrichr. Moreover, when comparing two functional enrichment analysis tools, we found that Enrichr outperformed g: Profiler by a significant margin. This finding suggests that the databases employed in Enrichr and cGSA are more effectively aligned with the requirements of context-aware gene set analysis, yielding better interpretability and precision than those used in g: Profiler. Due to its higher HIT rate and context-aware filtering, cGSA also outperforms other LLM-based GSA methods, though they generate a similar number of pathways per DEG set.

To align with common research practice, in which validation is often streamlined by prioritizing the most enriched pathways, we further compared cGSA with top *K* results (K=10,30,50) of functional enrichment analysis tools. As shown in Fig. 3c, cGSA outperforms Enrichr by 6% and g: Profiler by 11% at K=50, and respectively by 8% and 27% at K=10. Moreover, the HIT values of both Enrichr and g: Profiler peak only when the candidate set expands to 50 pathways (Supplementary Appendix B). These results indicate that many contextually relevant pathways are ranked lower in conventional enrichment outputs, requiring extensive manual inspection to avoid omission. By comparison, cGSA more effectively prioritizes context-relevant pathways within a compact result set, substantially reducing manual review effort.

### 3.4 High consistency between automatic and expert annotation

To assess the validity of similarity-based automatic evaluation, we recruited two domain experts to examine the functional associations between 1482 cGSA-generated pathways and 1,671 ground-truth pathways across 102 manually curated DEG sets. The experts achieved an inter-annotator agreement of 90.0%. Manual annotation was limited to cGSA-generated pathways, rather than outputs from competing methods, due to the high cost of expert review.

**Figure 4:**
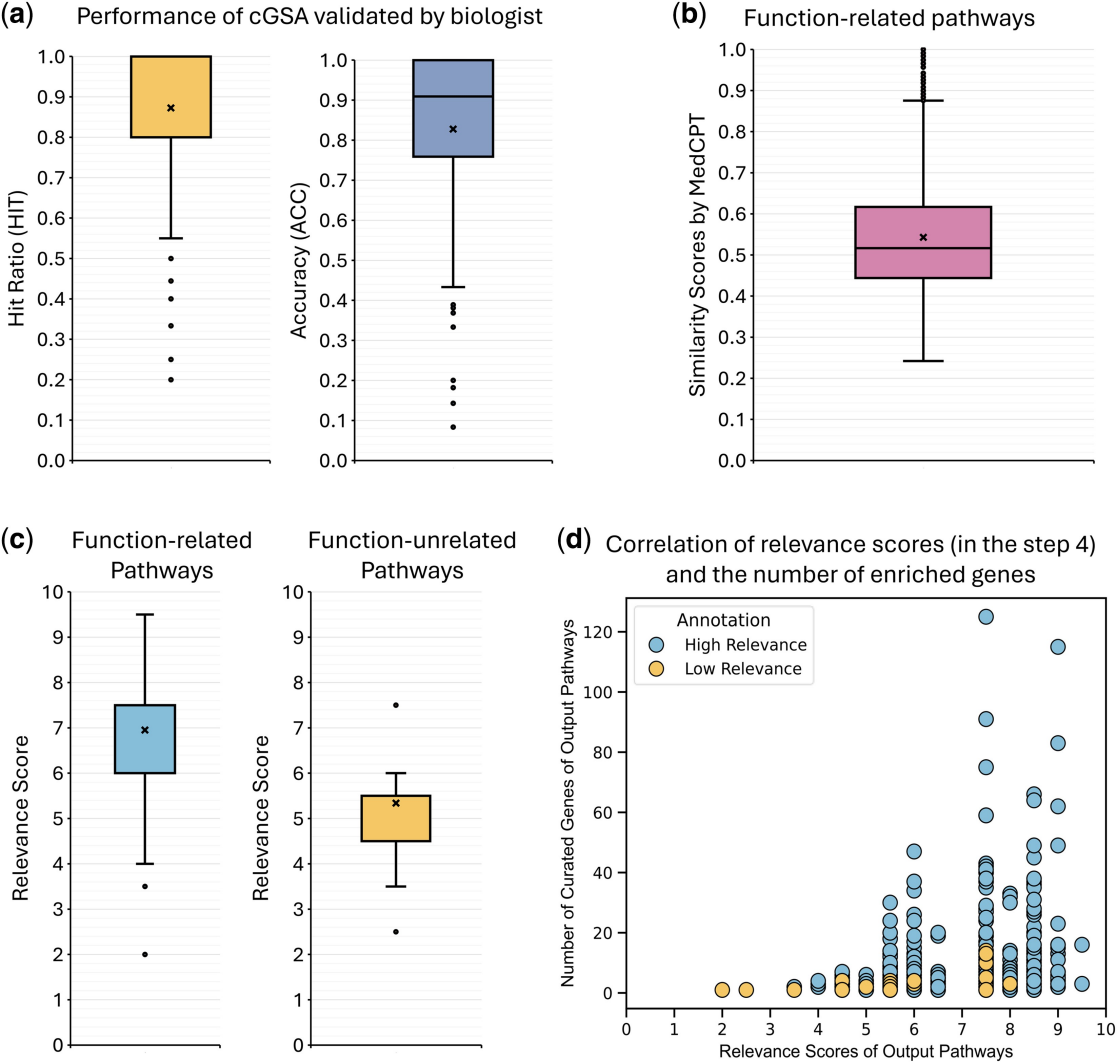
Expert annotation analysis of pathways identified by cGSA. (a) The calculation of HIT and ACC for output pathways with the same functional category as the ground truths, i.e. function-related pathways. HIT represents the proportion of ground truths covered by function-related pathways, while ACC denotes the proportion of function-related pathways in all identified pathways. (b) The distribution of semantic similarity scores for function-related pathways, as computed by MedCPT. (c) The relevance scores of function-related pathways compared to function-unrelated pathways to the context, where the context refers to the research topic. (d) Correlation between relevance scores of identified pathways and the number of their enriched genes curated in the database.

The human validation shown in Fig. 4a demonstrates that cGSA achieved a harmonic mean value of 0.850 (0.828 ACC and 0.873 HIT), which is closely alignment with the result of 0.879 obtained with the similarity score threshold of 0.5 (Fig. 3a). To further validate the reliability of 0.5 similarity threshold, we computed the MedCPT score for all functionally related pathways annotated by the human experts. This calculation yielded an average similarity of 0.543 (Fig. 4b), further supporting the use of the 0.5 threshold in the previous automatic method comparison analysis.

Additionally, we also compared relevance scores between functionally related and unrelated pathways. As shown in Fig. 4c, the related pathways achieved a higher average score (7.0) than unrelated pathways (5.3), which indicates that scores above 7.0 strongly reflect contextual relevance. We further analyzed the relationship between relevance scores and the number of curated genes annotated for each pathway (Fig. 4d), observing that pathways with fewer curated genes generally received lower scores (below 5.5), whereas pathways with larger curated genes tended to receive higher scores (above 5.5). Based on these observations, we defined two relevance thresholds: 5.5 for moderate contextual relevance and 7.0 for high contextual relevance.

### 3.5 Relevance score calibration

**Figure 5:**
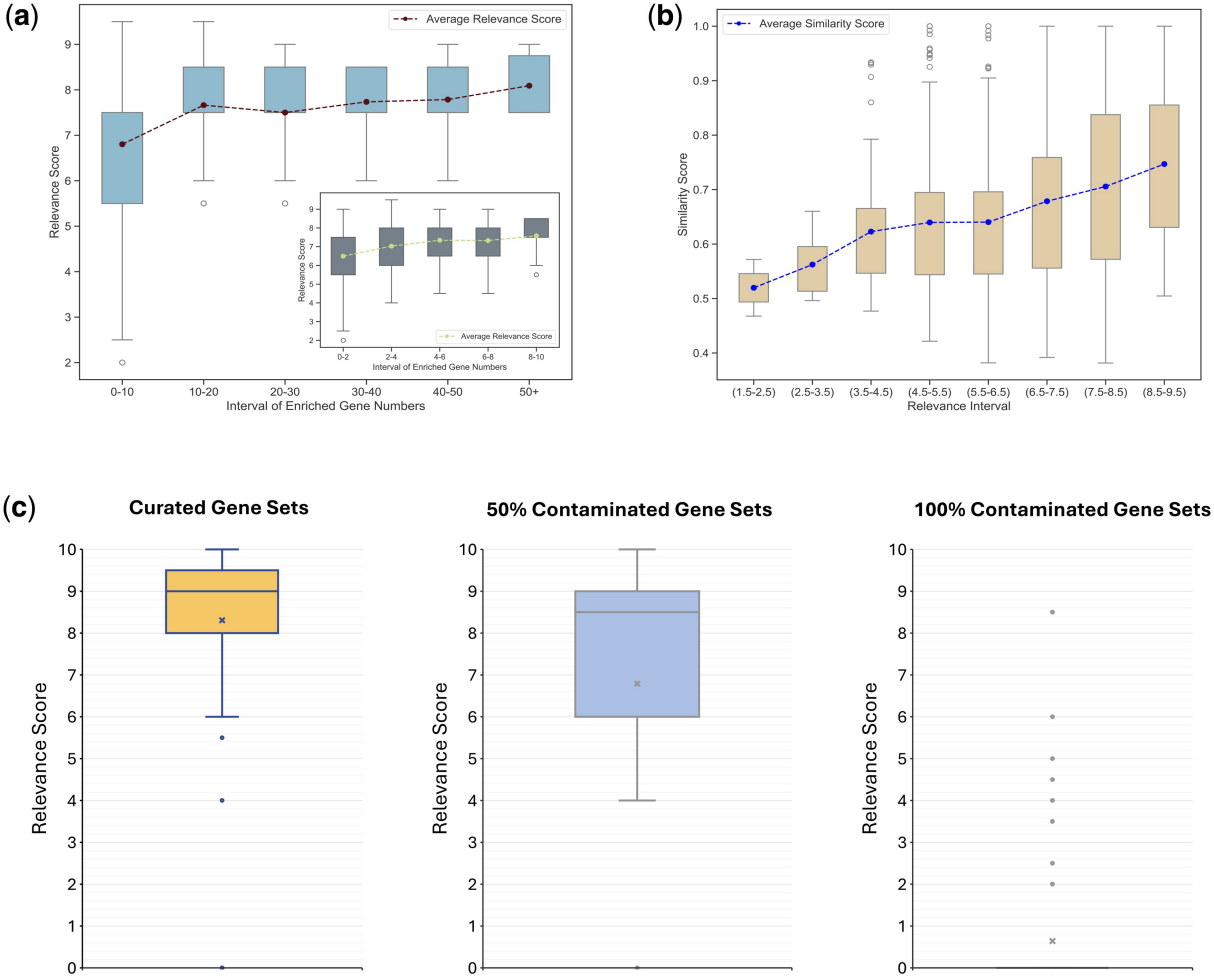
Relevance score calibration results. Shown are the correlations between relevance scores generated by cGSA and (a) the number of enriched genes in ground-truth pathways curated in databases, and (b) the semantic similarity scores computed by MedCPT between cGSA outputs and ground truths. In both analyses, the number of enriched genes and the relevance scores of all output pathways were grouped into discrete intervals along the x-axis. (c) Relevance scores for curated gene sets from Gene Ontology and their corresponding contaminated gene sets.

To further examine the confidence of relevance scores assigned by cGSA in assessing contextual relevance, we compare their correlation with two independent metrics: (1) the number of enriched genes curated in databases, shown in Fig. 5a, and (2) the semantic similarity scores computed by MedCPT between cGSA outputs and ground-truth annotations, shown in Fig. 5b.

Both analyses exhibit a monotonic upward trend, with the number of enriched genes and semantic similarity scores increasing as relevance scores rise. On the one hand, the number of genes overlapping curated enrichment results is a widely used indicator of functional significance in gene set enrichment and Gene Ontology annotation tasks. The observed trend in Fig. 5a therefore suggests that higher relevance scores correspond to stronger functional associations between predicted pathways and gene clusters. On the other hand, ground-truth pathways have been validated as contextually relevant in their original studies, implying that greater semantic similarity between cGSA outputs and ground truths reflects higher contextual relevance. Consistent with this expectation, the positive correlation shown in Fig. 5b demonstrates that higher relevance scores are associated with an increased likelihood of pathways being contextually relevant. These findings suggest that the relevance scores assigned by cGSA align well with independent similarity assessments, supporting their reliability in evaluating pathway-context relationships.

Additionally, to validate the robustness of the structured scoring prompt used in cGSA, we evaluated its ability to assess contextual relevance using curated and contaminated gene sets. The gene sets were obtained from the study by Hu et al. (2025), while contextual descriptions were derived from GO term definitions retrieved via the QuickGO API.^4^ Specifically, we collected 100 GO terms along with their associated gene sets as the curated gene sets (Fig. 5c). For each GO term, we generated a partially contaminated gene set in which 50% of genes were retained from the original GO annotation and the remaining 50% were randomly sampled from a background pool of genes with GO annotations (50% contaminated gene sets; Fig. 5c). We further constructed a fully randomized variant by sampling 100% of genes from the background pool (100% contaminated gene sets; Fig. 5c).

The results demonstrate that curated gene sets achieved the average relevance score of 8.31, exceeding the average score of 6.79 for 50% contaminated gene sets and substantially outperforming the average score of 0.64 for 100% contaminated gene sets. These findings confirm that the scoring prompt can effectively capture contextual relevance by distinguishing between biologically coherent and perturbed gene sets: *The moderate score reduction observed for partially contaminated gene sets indicates that the introduction of unrelated genes weakens contextual specificity, whereas the pronounced decline for fully randomized sets demonstrates that the absence of coherent biological structure results in minimal contextual relevance*.

### 3.6 cGSA is robust with different configurations

To evaluate the contribution of individual components in cGSA, we conducted a series of ablation experiments examining gene cluster detection, the choice of backend LLM, and the sensitivity of cGSA to context length.

#### 3.6.1 Gene cluster detection

**Figure 6:**
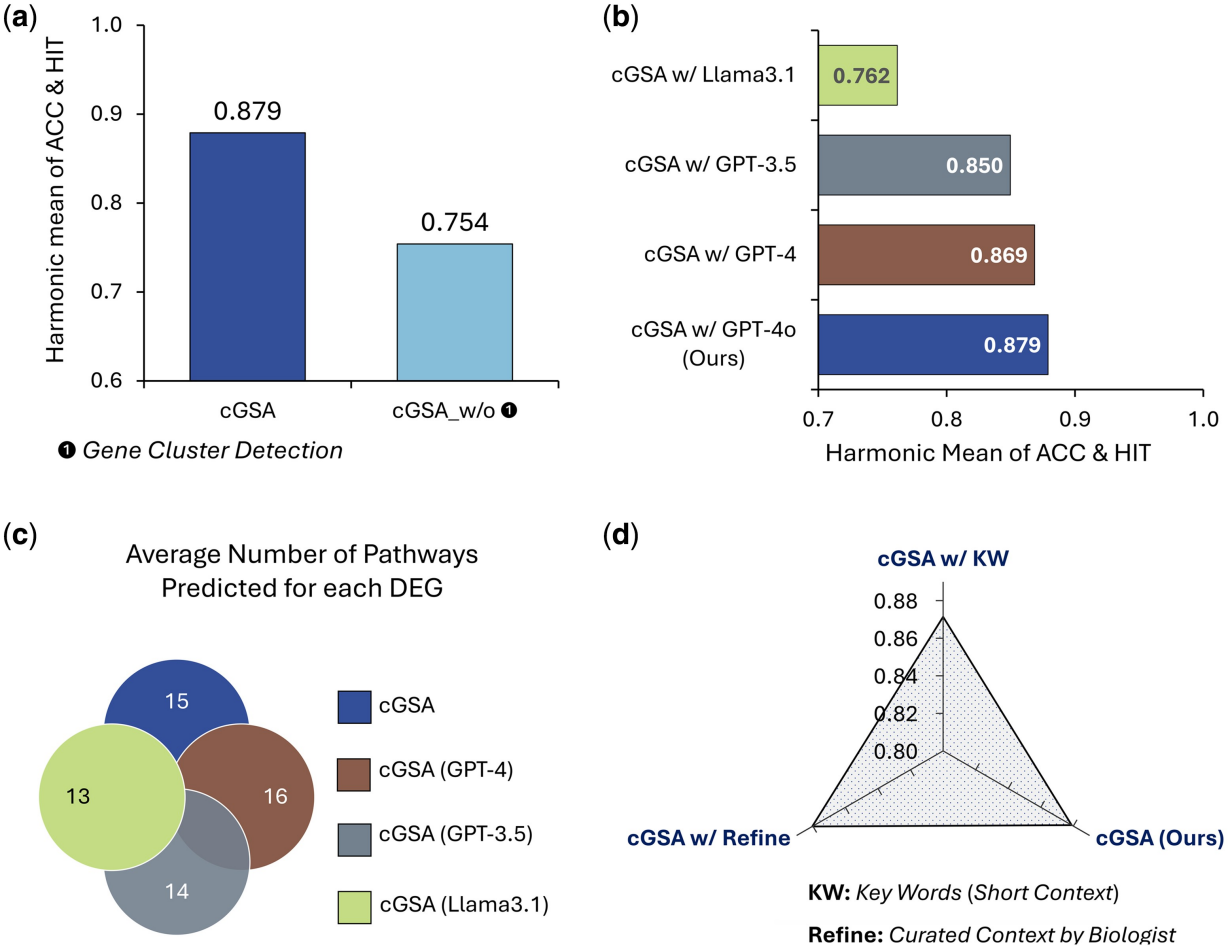
Ablation experiments for cGSA with a similarity score threshold of 0.5. (a) Comparison between cGSA and its variant without the Gene Cluster Detection step. (b) Performance comparison of cGSA using different large language models (LLMs). (c) The average number of output pathways per DEG identified by cGSA using different LLMs. (d) Performance comparison of cGSA across different research topic contexts. “KW” refers to keywords (2.59 averaged words) summarized from the context used in cGSA, while “Refine” represents the context manually curated by the biologist (8.71 averaged words) based on the original context used in cGSA.

To investigate the role of gene cluster detection in cGSA, we ablated this component and instead prompted the LLM to summarize context-related pathways based on enrichment analysis of the entire DEG set. As shown in Fig. 6a, the removal of gene cluster detection resulted in a 12.5% decrease. An additional ablation experiment on adding gene cluster detection to Enrichr show that the performance of Enrichr improves 2.1% (Supplementary Appendix D). These results demonstrate that configuring the gene cluster detection step substantially improves ground-truth coverage by identifying distinct gene subsets within the input DEG and mitigating inflated enrichment driven by central hub genes.

For example, in the analysis of DEGs from pancreatic tumor, cGSA without gene cluster detection produced “Complement,” “KRAS.KIDNEY UP.V1 UP,” “KRAS.50 UP.V1 UP,” and “KRAS.300 UP.V1 UP.” Despite differences in naming, these pathways are only involved in two functional categories: complement activation and KRAS signaling, which reflects redundancy caused by the disproportionate influence of the hub genes SCG5 and SCG3. In contrast, cGSA configured with gene cluster detection identified three contextual pathways: “mTORC1 signaling pathway,” “KRAS signaling pathway,” and “Oxidative Phosphorylation.” These results not only preserved the key biological signal of KRAS signaling but also captured a broader set of ground-truth pathways, yielding a higher HIT score and improved biological interpretability.

#### 3.6.2 LLM backend

We further evaluated the impact of different LLMs (GPT series vs. Llama) on the pathway screening and summarization steps. As shown in Fig. 6b and c, cGSA configured with GPT models demonstrated superior performance in summarizing context-aware pathways compared to Llama 3.1-70B. This effect arises from differences in response behavior between GPT and Llama models when using the fully engineered prompt. The GPT models prioritize summarizing a single prominent functional term, but Llama models like to concatenate multiple significant pathways. For instance, Llama model generates the term “Regulation of Cell Migration and Angiogenesis” for DEGs derived from a melanoma-related study, whereas the GPT model summarized it as “Regulation of MAPK Cascade.” The latter exhibits greater semantic relevance to the ground-truth annotation, highlighting GPT’s tendency to produce more contextually precise pathway interpretations.

Additionally, the harmonic mean value of ACC and HIT showed no significant difference between cGSA using GPT-4o and GPT-4. cGSA performance was also consistent when comparing DEGs published in 2024 with those from 2022 to 2023. These results suggest that cGSA is not highly sensitive to the knowledge cutoff differences among GPT-4 versions, because the initial gene cluster detection step effectively partitions input DEGs into previously uncharacterized gene subsets using the EdMot community detection method.

#### 3.6.3 Different phrases of contextual terms

To further examine the impact of context length on pathway summarization, we evaluated cGSA using three context lengths as the inputs. In addition to the original research contexts extracted from published articles (average length: 6.92 words), we generated concise keyword-based contexts (average length: 2.59 words) and extended professional statements (average length: 8.71 words). For instance, a research objective context described in the original study was “pancreatic neuroendocrine tumors” while the reduced keyword phrase was “pancreatic neuroendocrine” and the expanded professional statement was “clinically aggressive and nonaggressive proteome subtypes of pancreatic neuroendocrine tumors.” As shown in Fig. 6d, the harmonic mean scores range from 0.871 to 0.880 across these settings, indicating that cGSA maintains robust performance across varying context lengths, including very short contextual inputs.

### 3.7 Real-world case studies using cGSA

#### 3.7.1 Analysis for adaptive DEGs in melanoma evolution

**Figure 7:**
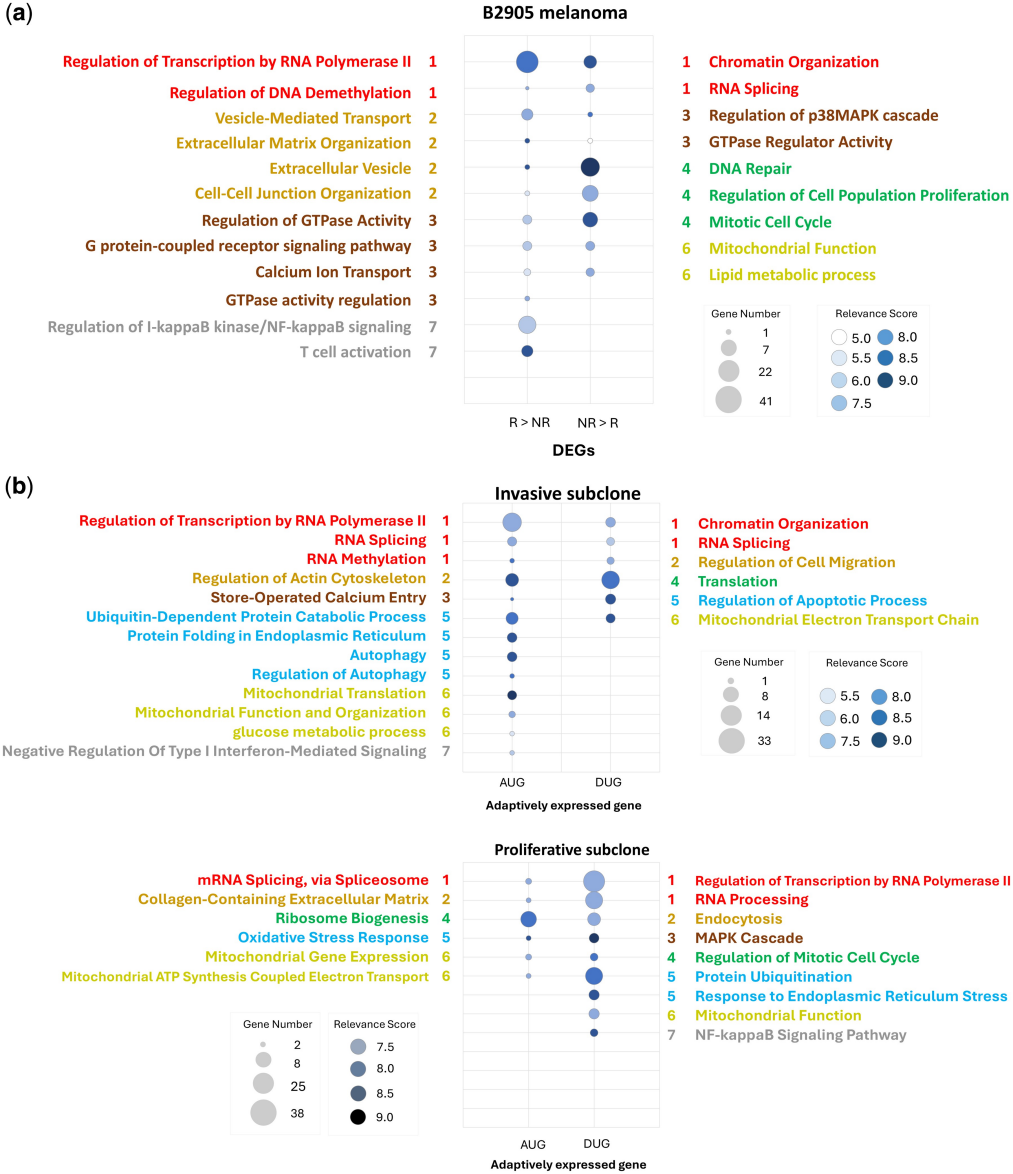
Case studies for melanoma cancer. (a) Pathways identified by cGSA as enriched in DEGs between responding (R) and non-responding (NR) B2905 melanoma samples treated with anti–CTLA-4 immune checkpoint blockade (ICB) therapy. (b) Pathways identified by cGSA as enriched in adaptively upregulated genes (AUGs) and adaptively downregulated genes (DUGs) from the invasive subclone group (top) and the proliferative subclone group (bottom).

In a recent study, Hirsch et al. (2025) identified the adaptively up- and down-regulated genes (AUGs and DUGs, respectively) in two specific subclone groups out of 24 single cells isolated from a parental mouse melanoma B2905 cell line, one group exhibiting an invasive phenotype and the other a proliferative phenotype. To validate the utility of cGSA in such studies, we applied the pipeline to analyze DEGs between responding (R) and non-responding (NR) B2905 tumors receiving ICB treatment in a preclinical study (Fig. 7a) as well as the AUGs and DUGs of the subclone groups with invasive and proliferative phenotype information (Fig. 7b).

As shown in Fig. 7, we categorized the pathways into seven functional groups and color-coded them accordingly. Pathways enriched in genes with higher expression in responding tumors (R>NR) were primarily dominated by the categories 1 and 7, respectively corresponding to transcriptional regulation and inflammatory responses. While the enrichment in the categories two and three reflect biological processes related to cell migration and intracellular signaling. Pathways enriched in genes with higher expression in non-responding tumors (NR>R) were associated with functions related to cell cycle. Comparing these pathways with those identified in invasive subclone groups revealed substantial functional overlap, such as the process of “regulation of transcription by RNA polymerase II” and “chromatin organization.” This observation suggests that categories 1 and 2 represent shared phenotypic characteristics of immune checkpoint blockade (ICB)–resistant subclones. This observation is consistent with prior studies reporting that therapeutic resistance in melanoma is associated with transcriptional reprogramming toward mesenchymal-like phenotypes (Arozarena and Wellbrock 2019). In contrast, pathways related to the cell cycle dominated the enrichment of adaptively upregulated genes (AUGs) in proliferative subclone NR>R genes, indicating a selective advantage of proliferative phenotypes in the absence of effective therapy-induced immunity.

As Hirsch et al. merely focused on genes exhibiting adaptive expression, cGSA further extends the findings by elucidating the functional roles of genes under in vivo selection pressure. Notably, although similar functional pathway categories were identified across subclone groups and ICB-treated tumors, the underlying gene sets showed minimal overlap. This result highlights cGSA’s ability to perform higher-level functional interpretation of gene sets, rather than relying solely on direct statistical overlap with predefined databases.

#### 3.7.2 Cell–cell communication within the breast cancer tumor microenvironment

Cell–cell communication (CCC) within tumor microenvironment (TME) plays a critical role in regulating immune responses (Lv et al. 2023, Griffiths et al. 2025). To evaluate the applicability of cGSA for analyzing CCC within the TME, we applied the pipeline to DEGs identified from on-treatment versus pre-treatment comparisons across T cells, myeloid cells, and endothelial cells. These DEGs were derived from single-cell RNA-sequencing data from 31 breast cancer patients treated with anti–PD-1 therapy (Bassez et al. 2021).

**Figure 8:**
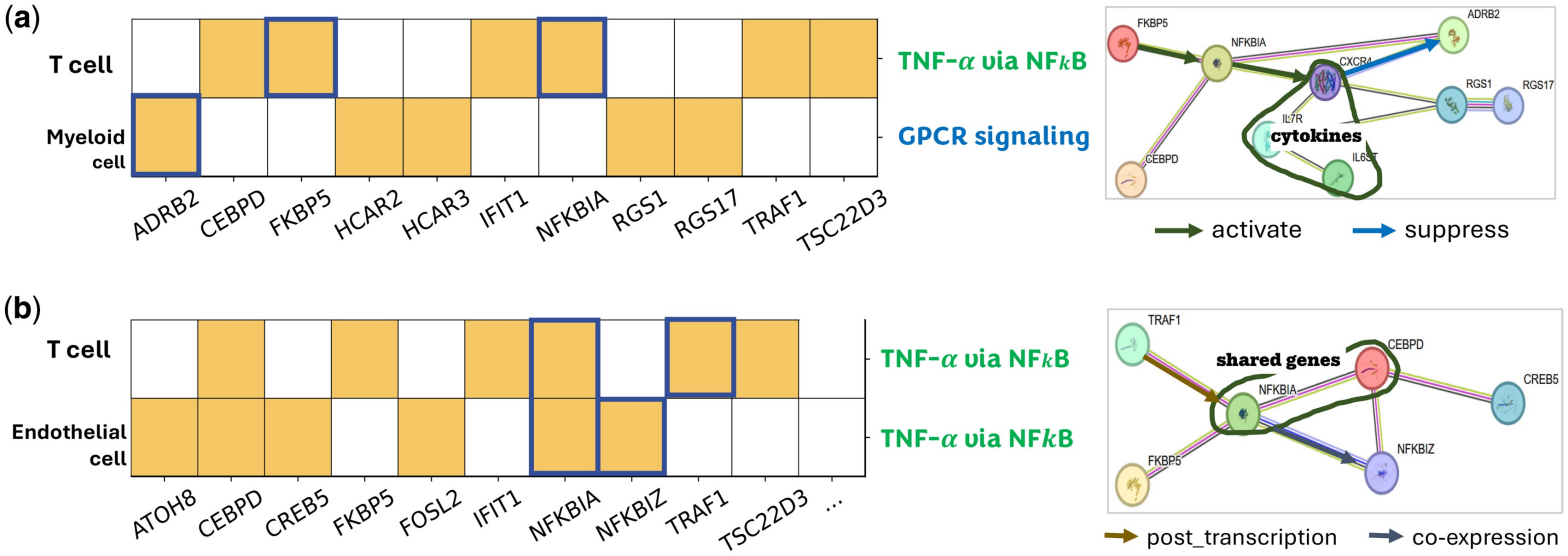
Case study of cell–cell communication within the TME. Shown are representative pathways and associated genes identified by cGSA from differentially expressed genes (DEGs) in T cells, myeloid cells, and endothelial cells. Gene interactions are derived from the protein–protein interaction (PPI) network (https://string-db.org).

As shown in Fig. 8a, our result suggests that FKBP5 is involved into the TNF-α signaling pathway (via NF-κB) in T cells, whereas ADRB2 is involved in the GPCR-cAMP signaling pathway in myeloid cells. This observation aligns with reports that NF-κB inflammatory signaling is enhanced in T cells during effective anti-PD1 therapy and that FKBP5 can potentiate NF-κB activation in inflammatory and cancer contexts, ultimately leading to upregulation of FKBP5 in pro-inflammatory states (Habara et al. 2022). FKBP5, in turn, facilitates sustained NF-κB activation by scaffolding IKK kinases and promoting NFKBIA turnover, with compensatory induction of NFKBIA as part of the canonical negative feedback loop (Jie et al. 2021). This enhances the release of pro-inflammatory cytokines including TNF-α and Interleukins via the cytokine signaling pathway, contributing to an inflammatory microenvironment in which ADRB2 expression is reduced. The decreased ADRB2, which normally exerts immunomodulatory effects, is consistent with a shift toward a more pro-inflammatory myeloid phenotype (Stolk et al. 2023).

As shown in Fig. 8b, cGSA suggests a causal coordination of inflammatory signaling between T cells and endothelial cells in the immune response, mediated through the activation of TNF-α signaling via NF-κB. These two cell types share a key regulatory gene, NFKBIA, which acts as a downstream effector of TRAF1 signaling in T cells and is co-expressed with the gene NFKBIZ in endothelial cells. This finding aligns with prior studies showing that anti-PD1 treatment activates T cells, leading to the upregulation of TRAF1 and modulation of NF-κB signaling via NFKBIA (Li and Verma 2002). Activated T cells secrete TNF-α, which binds to TNFR1 on endothelial cells, triggering NF-κB activation and the induction of NFKBIZ expression (Bradley 2008). NFKBIZ then promotes the transcription of TNFAIP3 and NF-κB–dependent inflammatory mediators including endothelial-activation genes like CYR61, thereby driving endothelial activation (You et al. 2014). These activated endothelial cells then recruit immune cells to sustain T-cell activation and cytokine secretion, thereby establishing a positive feedback loop that amplifies the immune response (Meng et al. 2024).

In conclusion, cGSA reveals gene communications through functionally similar pathways, which is particularly valuable in immunotherapy-related studies, where multiple immune and stromal cell populations interact through complex signaling networks.

## 4 Conclusion

While cGSA demonstrates strong performance in identifying contextually relevant pathways, it may occasionally generate pathways with low contextual relevance. This limitation is likely due to the limitations of the gene cluster detection module, particularly when relying solely on the PPI network. To address this issue, future iterations of cGSA could benefit from integrating gene regulatory networks to enhance gene clustering. Our current benchmarking focuses on human and mouse DEG sets, as these are the most widely represented species in GSEA databases and remain central to functional genomics research. However, expanding support for additional species represents a valuable direction for future work.

## Supplementary Material

btag214_Supplementary_Data

## Data Availability

The demo website is publicly available at https://www.ncbi.nlm.nih.gov/CBBresearch/Lu/Demo/cGSA/, while the data and code can be accessed at https://github.com/ncbi-nlp/cGSA.

## Footnotes

1
https://string-db.org/help/api/

2
https://maayanlab.cloud/Enrichr

3
https://www.ebi.ac.uk/ols4/api-docs

4
https://docs.identifiers.org/pages/api.html
