# Supplementary material for: Knowledge-guided contextual gene set analysis with large language models
Source: Bioinformatics. 2026 Jul 7;42(Suppl 1):btag214. doi: 10.1093/bioinformatics/btag214 (PMC13340159; doi:10.1093/bioinformatics/btag214)
Supplement: btag214_Supplementary_Data [file btag214_supplementary_data.pdf]

A. Structured Prompts in cGSA

A.1 Prompts used for pathway screening

System

You are an efficient evaluator for the pathway and the related context of experimental conditions.

User

Give me a confidence score within 0-10 (e.g., 8.25) to evaluate the relevance of pathways to the biological context **#\n{context}\n#**.

The confidence score is used to denote how the important does the pathways related to experimental conditions of the given gene set.

You should evaluate this relevance based on two criteria.

(1) Pathway analysis: Analyzing the curated functions of the pathway to explore whether it is directly or indirectly associated with the disease. The directly relevance should have a higher score while the indirectly relevance should have a lower score. If there is no association between pathway and disease, the confidence score should be absolutely 0.00.

Here is the detail of enriched pathway: **#\n{name}\n#**

Here is the function of the pathway: **#{definition}#**

(2) Gene function exploration: Considering the functions of every gene and investigate how many genes can be involved into the background context. Also explore whether there are famous or well-known genes to the background context. If so, adjust the score higher appropriately.

Here are the genes: **#\n{genes}\n#**

Based on your score, decide whether the given pathway is related to the context or not.

A.2 Prompts used for pathway summarization

There are two instructions in the pathway summarization step: one is used for summarizing the prominent pathway from all candidate enriched terms, while the another is used for scoring the summarized pathway to indicate the contextual relevance.

System

You are an efficient assistant for biologists to explore the representative pathways of the interacting genes based on the context.

User

I have obtained some genes, which are functional related to the experimental conditions and configurations.

I also obtained some candidate biological functions involving pathways, GO terms, disease ontologies, etc., which are enriched from the biological databases. Your task is to summarize the most representative biological function of the interacting genes based on the given experimental context. Let's think step by step to complete this task: **First, categorize those candidates with the similar biological mechanism together based on the experimental context. Second, write a critical analysis for every category based on the provided analysis of each function to summarize the most representative function name. Third, synthesize function names of different categories and propose the most significant name associated with the experimental context. Last, write a critical analysis for the interacting genes and investigate whether the final proposed name can be supported by the gene functions.**

For the proposed name, its biological functions must be very related to the context. It also should be concise and close to the phrase of the enriched terms as much as possible. To this end, you can use the gene ontology term to adjust the proposed name.

I will give you one example for such a task: **For the interacting genes: CCNB1,RRM2B,PPP2R1B,BIRC5,CDC16,RRM2,BORA,NUF2, they are overlapped with the pathways that are related to "Enrichment analysis for genes of melanoma disease": cell cycle; cell cycle, mitotic; mitotic prometaphase. These pathways can be categorized as "cell cycle; cell cycle, mitotic;" and "mitotic prometaphase; cell cycle, mitotic" based on their enrichment results. These two categories are summarized as "cell cycle" and "mitotic" that match the "cell cycle, mitotic" in the enrichment analysis, so that the proposed pathway name can be "mitotic cell cycle", which is also indeed related to the context. After this, the biological process analyzed from the interacting genes is "cell cycle", which is very similar to the proposed name. By considering the context again, the final pathway name is confirmed as "mitotic cell cycle".**

Here are the genes: **#\n{genes}\n#**

Here is the experimental context: **#\n{context}\n#**

Here are candidate terms and their corresponding information (separated by ;): **#\n{pathways}\n#**

Here are functional descriptions of these genes: **#\n{summary}\n#**

System

You are an efficient assistant for a biologist to evaluate the relevance between a pathway and the background context.

User

I have obtained the significant pathway of a list of genes. Here it is: **#{pathways}#**

Write a critical analysis for the pathway to explore its relevance to the background topic. Here is the background topic: **#{context}#.**

After completing your analysis, assign a relevance score (with two decimal places) to the pathway based on the given topic. Notice that this score helps gauge how much does the pathway functions and their corresponding genes be related to the background context. This score should range in [0.00, 10.00]. A score of 0.00 indicates the lowest relevance possibility, while 10.00 reflects the highest relevance possibility.

This score should be based on the comprehensive consideration of the following steps:

1. Understand the background topic and identify all explicit and implicit biological mechanisms relevant to it. 2. Analyze the pathway's functional roles by examining all functions represented within the pathway that relate to any mechanisms of the background topic and assess the significance of these functions. 3. Review the strong evidence in the provided articles (if available) and evaluate the strength of associations between the background topic and the pathway. 4. Identify all potential functional associations between the pathway and the background topic and quantify how relevant these associations are.

Only give a float number of score, and don't generate any other analysis texts.

There are some retrieved PubMed articles to help you discover the potential associations between the pathway and the background topic. Here are the articles: **#{abstract}#**

Provide a comprehensive and informative analysis explaining how the proposed pathway is related to the background topic. You must explicitly cite the correct PMIDs whenever you reference conclusions from the provided articles. Use the citation format: "<conclusion> (PMID: xxx)".

B. Details of Data Collection for DEG Curation

Following the general study pipeline for genomic research, the articles retrieved for benchmark curation includes four key factors: (1) Research objectives (e.g., Impact of Threonine 4 phosphorylation of RNA Pol II on gene expression); (2) Experimental conditions (e.g., negatively regulated genes upon 52X T4A-CTD expression); (3) Selected functions/pathways, which are usually presented in figures or tables in the main text; and (4) DEG sets, which is usually archived in the supplementary tables.

Based on these four criteria, we queried PubMed using the search term "Gene Set Enrichment Analysis (GSEA)" and restricted the publication timeline to between October 2022 and June 2024. The scope was further narrowed to articles in the PMC Open Access Subset or those including supplementary tables. We then applied GNorm2 to recognize gene mentions in the supplementary tables and count their frequencies. Only articles with at least 50 genes in the supplementary tables were selected. In total, 331 articles met the criteria and were available for manual curation. Over four weeks, we carefully reviewed each DEG set along with the corresponding research objectives, experimental conditions, and manually selected functions or pathways described in each article. Ultimately, we curated 102 DEG sets from 31 articles as ground-truth functions.

C. ACC and HIT of cGSA and Other baselines

The detailed ACC and HIT scores of cGSA and the baseline method, as well as the variants of classical enrichment analysis tools are shown in Tab.1T̃ab.4. More detailed data related to ACC and HIT can be accessed at `https://github.com/ncbi-nlp/cGSA`.

Table 1. Accuracy (ACC) of different models.

Models	> 0.5	> 0.6	> 0.7	> 0.8	> 0.9
cGSA	0.913	0.616	0.347	0.236	0.165
Enrichr	0.778	0.419	0.212	0.111	0.011
g:Profiler	0.515	0.271	0.123	0.056	0.025
GPT-4o	0.934	0.660	0.392	0.190	0.096
GPT-4	0.940	0.660	0.354	0.126	0.069
Llama3.1	0.941	0.659	0.388	0.171	0.076

Table 2. Hit ratio (HIT) of different models.

Models	>0.5	>0.6	>0.7	>0.8	>0.9
cGSA	0.845	0.590	0.371	0.227	0.130
Enrichr	0.897	0.708	0.534	0.316	0.063
g:Profiler	0.638	0.449	0.282	0.175	0.088
GPT-4o	0.345	0.273	0.172	0.099	0.050
GPT-4	0.336	0.263	0.163	0.097	0.040
Llama3.1	0.385	0.305	0.200	0.103	0.053

D. Ablation Experiment for Gene Cluster Detection

Fig.1 shows the results of Enrichr configured with gene cluster detection, which improves performance by 2.1% compared

Table 3. Accuracy (ACC) of cGSA and top-K results from classical enrichment analysis.

Models	>0.5	>0.6	>0.7	>0.8	>0.9
cGSA	0.913	0.616	0.347	0.236	0.165
g:Profiler (top-50)	0.381	0.202	0.093	0.045	0.022
Enrichr (top-50)	0.801	0.461	0.241	0.130	0.017
g:Profiler (top-30)	0.335	0.179	0.084	0.042	0.021
Enrichr (top-30)	0.807	0.480	0.258	0.144	0.018
g:Profiler (top-10)	0.204	0.105	0.048	0.027	0.014
Enrichr (top-10)	0.825	0.537	0.317	0.188	0.024

Table 4. Hit ratio (HIT) of cGSA and top-K results from classical enrichment analysis.

Models	>0.5	>0.6	>0.7	>0.8	>0.9
cGSA	0.845	0.590	0.371	0.227	0.130
g:Profiler (top-50)	0.612	0.377	0.200	0.097	0.051
Enrichr (top-50)	0.896	0.693	0.500	0.286	0.055
g:Profiler (top-30)	0.595	0.347	0.178	0.084	0.041
Enrichr (top-30)	0.876	0.639	0.451	0.258	0.046
g:Profiler (top-10)	0.499	0.248	0.113	0.055	0.025
Enrichr (top-10)	0.766	0.465	0.293	0.167	0.023

to the classical setting. These results demonstrate that gene cluster detection helps mitigate inflated enrichment driven by central hub genes.

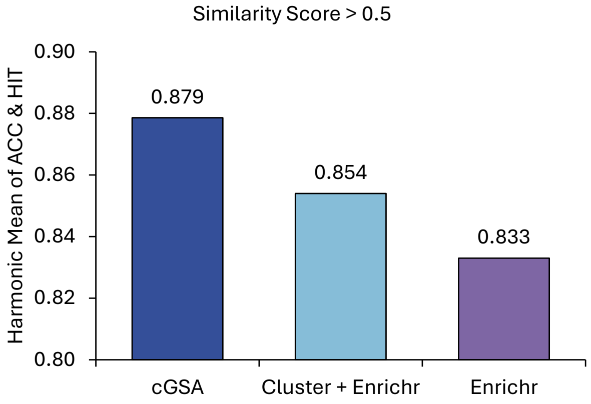

Fig. 1. The results of adding gene cluster detection to Enrichr.
